# Data on the involvement of Meox1 in balloon-injury-induced neointima formation of rats

**DOI:** 10.1016/j.dib.2017.11.061

**Published:** 2017-11-22

**Authors:** Bing Wu, Lei Zhang, Yun-He Zhu, You-en Zhang, Fei Zheng, Jian-Ye Yang, Ling-Yun Guo, Xing-Yuan Li, Lu Wang, Jun-Ming Tang, Shi-You Chen, Jia-Ning Wang

**Affiliations:** a Institute of Clinical Medicine and Department of Cardiology, Renmin Hospital, Hubei University of Medicine, Shiyan, Hubei 442000, People's Republic of China; b Key Lab of Human Embryonic Stem Cell of Hubei Province and Department of Physiology, Hubei University of Medicine, Hubei 442000, China; c Department of Nuclear Medicine, Renmin Hospital, Hubei University of Medicine, Shiyan, Hubei 442000, People's Republic of China; d Department of Physiology & Pharmacology, The University of Georgia, Athens, GA 30602, USA

**Keywords:** Meox1, SMCs, Balloon-injury, Neointimal formation

## Abstract

In the previous report, Meox1 was found to promote SMCs phenotypic modulation and injury-induced vascular remodeling by regulating the FAK-ERK1/2-autophagy signaling cascade (Wu et al., 2017) [1]. Here, we presented new original data on the involvement of Mesoderm/mesenchyme homeobox gene l (Meox1) in balloon-injury-induced neointima formation of rat. In rat carotid artery balloon injury model to induce vascular remodeling, Meox1 was induced in vascular smooth muscle cell (SMCs) of rat carotid arteries. Most proliferating cell nuclear antigen (PCNA)-positive cells also expressed Meox1. These data suggested that Meox1 may be involved in SMCs proliferation during injury-induced neointima formation. Furthermore, knocked down its expression in injured arteries by adenoviral delivery of Meox1 short hairpin RNA (shRNA) (shMeox1), neointima formation was significantly inhibited. Elastin staining also confirmed the reduction of neointima in Meox1 shRNA-transduced arteries. Moreover, knockdown of Meox1 decreased the collagen production/deposition that was significantly increased in neointima induced by balloon injury.

**Specifications Table**

**Table:** 

Subject area	Basic Medicine
More specific subject area	Vascular biology
Type of data	Figures and text
How data was acquired	The images were obtained from Microscope, Nikon 80i. Immunoblotting were measured with densitometry by Image J software (NIH).
Data format	Raw and analyzed
Experimental factors	The carotid arteries were fixed in 4% paraformaldehyde and embedded in paraffin, and then were cut into sections with 5 µm thickness.
	The segments for Western blotting were grinded in liquid nitrogen and homogenized on ice in RIPA buffer containing protease inhibitors.
Experimental features	H&E, Masson's, and Verhoeff's elastic staining were used to observe the morphology of the tissue. Western blot, IHC or IF were used to analysis the expression of Meox1 and PCNA. The immunoblots exhibited by enhanced chemiluminescence reaction were measured with densitometry by Image J software (NIH).
Data source location	Institute of Clinical Medicine and Department of Cardiology, Renmin Hospital, Hubei University of Medicine, Shiyan, Hubei 442000, People's Republic of China;
Data accessibility	The data are available with this article
Related research article	Mesoderm/mesenchyme homeobox gene l promotes vascular smooth muscle cell phenotypic modulation and vascular remodeling (DOI: 10.1016/j.ijcard.2017.10.098)

**Value of the data**

1) The data revealed that Meox1 was induced in arterial SMCs during injury-induced vascular remodeling, and promoted injury-induced neointimal formation in rat carotid artery.
2) These data are the further evidence on the interplay between neointimal formation and Meox1 [1].
3) The data in this article provide the potential therapeutic effect of Ad-Sh-Meox1 on restenosis following percutaneous coronary intervention (PCI) through promoting re-endothelialization while inhibiting neointimal formation.

## 1 Data

**Fig. 1:**
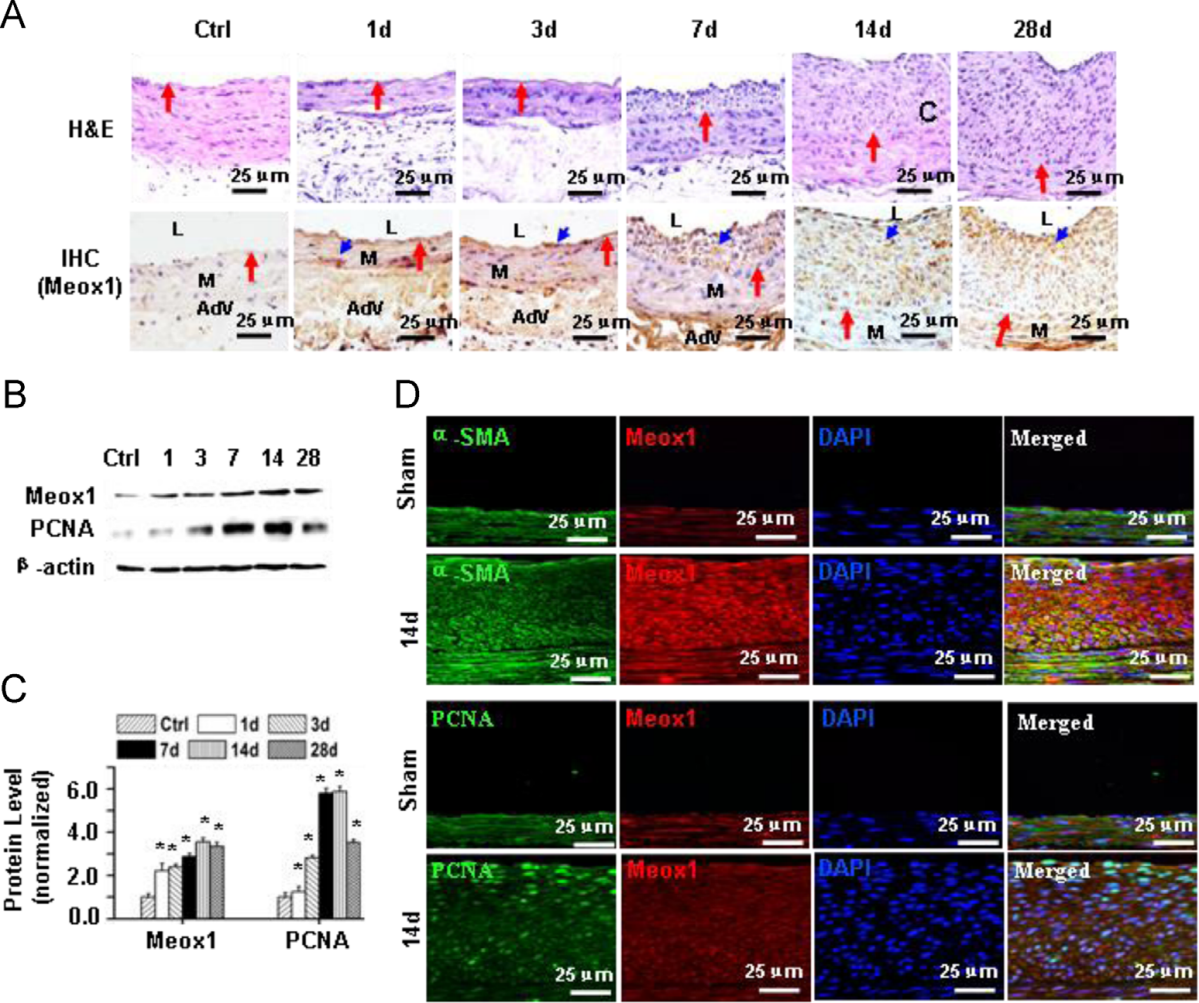
Balloon injury induced Meox1 expression in SMCs of rat carotid arteries. A, Meox1 was induced initially in medial SMC and subsequently in neointima SMCs in carotid arteries following Balloon injury. Red arrows indicate internal elastic laminae; blue arrows show Meox1-positive cells. M: medium; L: lumen; Adv: adentitia. B, Balloon injury induced a time-dependent expression of Meox1 and PCNA proteins in carotid arteries as detected by Western blotting. Data shown are representative results of 3 independent experiments. C, Quantification of protein levels shown in B by normalizing to β-actin. *P≤0.05 compared with the sham group (Ctrl), *n*=3. D, Representative immunostaining images showing co-staining of Meox1 with α-SMA and PCNA in SMCs of injured arteries.

To determine the role of Meox1 in SMCs phenotypic modulation *in vivo*, we used rat carotid artery balloon injury model to induce vascular remodeling. Injury induced progressive neointima formation in the arteries (Fig. 1A). Meox1 was initially induced in medial SMCs (1 and 3 days after the injury) and subsequently in neointimal SMCs (7–28 days after the injury) (Fig. 1A). To quantify Meox1 expression, we performed Western blot using proteins extracted from the sham-operated control or balloon-injured arteries. As shown in Fig. 1B–C, Meox1 was time-dependently induced along with a cell proliferating marker PCNA in carotid arteries following injury. To determine if Meox1 was expressed in SMCs and if Meox1-expressing cells contributed to the SMCs proliferation, we co-stained Meox1 with smooth muscle α-actin (α-SMA) and PCNA in arteries injured for 14 days, respectively. As shown in Fig. 1D, most Meox1-expressing cells were α-SMA-positive. Most PCNA-positive cells also expressed Meox1 (Fig. 1D).

**Fig. 2:**
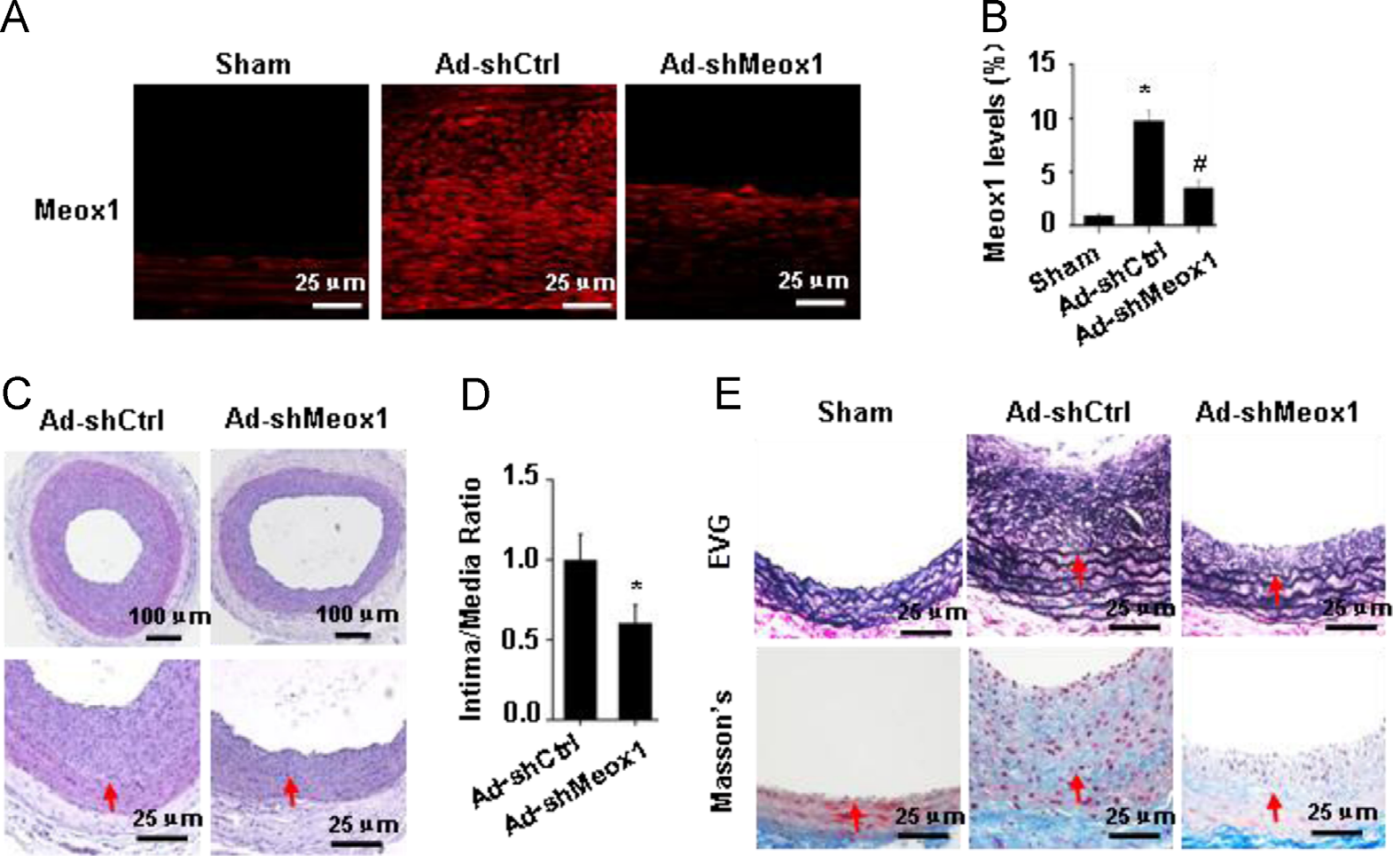
Knockdown of Meox1 attenuated balloon-injury-induced neointima formation. A, Meox1 shRNA delivery by adenovirus transduction (Ad-shMeox1) effectively knocked down Meox1 expression in balloon-injury-induced neointima SMCs. B, Meox1 levels were quantified by comparing the fluorescent immunostaining intensity (Sham set as 1). *P<0.05 vs the sham group (Ctrl); ^#^*P*<0.05 vs control adenovirus-transduced arteries (Ad-shCtrl), *n*=3. C–D. Knockdown of Meox1 attenuated neointima formation (C) and intima/media ratio (D) as shown by H&E staining. Red arrows: internal elastic laminae. *P<0.05 vs shCtrl-transduced arteries, *n*=5. E, Verhoeff's elastic stain (VEG) staining confirmed the effect of Meox1 knockdown on injury-induced neointima formation. Masson's staining showed that knockdown of Meox1 reduced the collagen deposition in neointima. Red arrows indicate internal elastic laminae.

To determine if Meox1 plays a role in the injury-induced neointimal formation, we knocked down its expression in injured arteries by adenoviral delivery of Meox1 shRNA. Knockdown of Meox1 (Fig. 2A–B) significantly inhibited the neointima formation as well as the intima/media ratio by nearly 40% (Fig. 2C–D). Elastin staining also confirmed the reduction of neointima in Meox1 shRNA-transduced arteries. Moreover, knockdown of Meox1 decreased the collagen production/deposition that was significantly increased in neointima by balloon injury (Fig. 2E), which is likely due to the role of Meox1 in modulating SMCs phenotype in the neointima.

## 2 Experimental design, materials, and methods

### 2.1 Rat carotid artery injury model

According to our previous protocals for model of balloon-injury carotid artery of rat [2], the mix gas including Isoflurane (1.5–2.5%) and oxygen was used for anesthetizing rats. After introducing a 2F Fogarty arterial embolectomy balloon catheter through the external carotid artery in left, pre-filled saline in the catheter were injected with 0.02 mL to inflate the balloon, and then rotatably withdrawn through the common carotid artery from proximal to the distal end of the heart. The above procedures were repeated for 3 times to achieve the perfect balloon-injury carotid artery with the traits of utter endothelial denudation. 100 µL of saline, Ad-Null or Ad-shMeox1 was injected into the balloon-injury carotid arteries through the specific syringe (WISP SYR, Hamilton), respectively. Subsequently, in order to transduce expression adenoviral vector of the interest genes into the cells of balloon-injury carotid artery, filling saline, Ad-Null or Ad-shMeox1 were resided in these arteries for incubation of 20 min, respectively.

### 2.2 Histomorphometric analysis and immunohistochemistry staining

The common carotid arteries were fixed in 4% paraformaldehyde and embedded in paraffin. The sections with 5 µm thickness were stained with modified hematoxylin and eosin (H&E), Masson's, and Verhoeff's elastic stain (VEG) Kit, respectively. For immunohistochemistry (IHC), sections with 5 µm thickness were rehydrated, blocked with 5% horse serum and incubated with a series of primary antibodies: goat anti-Meox1 (1:100, SC-10185, Santa Cruz Biotechnology), goat anti-Meox1 (1:200, ab105349, ABCAM), mouse anti-smooth muscle α-actin (α-SMA, 1:100, sc-130616, Santa Cruz Biotechnology), or proliferating cell nuclear antigen (PCNA) (1:200, ab29, Abcam) overnight at 4 °C followed by incubation with horseradish peroxidase. For immunoflurescent staining, fluorescein isothiocyanate (FITC) or tetraethyl rhodamine isothiocyanate (TRITC)-conjugated secondary antibody was used. The sections were counterstained with hematoxylin or 4,6-diamino-2-phenyl indole (DAPI).

### 2.3 Cell lysis, immunoblotting and antibodies

The segments for Western blotting were grinded in liquid nitrogen and homogenized on ice in Radio-Immunoprecipitation Assay (RIPA) buffer containing protease inhibitors. Cell or tissue debris was removed by centrifugation. 30 µg of proteins were separated in SDS-PAGE and were transferred to polyvinylidene difluoride membrane (Millipore). After being blocked by 5% fat free milk, the membrane was incubated with a series of primary antibodies: Meox1 (1:500, SC-10185, Santa Cruz Biotechnology), Meox1 (1:1000, ab105349, ABCAM), PCNA (1:500, sc-56, Santa Cruz Biotechnology), or β-actin antibody (1:5000, A5441, Sigma) followed by incubation with horseradish peroxidase-conjugated secondary antibodies (1:10000, Santa Cruz). The immunoblotsexhibited by enhanced chemiluminescence reaction (Amersham Pharmacia Biotech) were measured with densitometry by Image J software (NIH).

### 2.4 Statistical analysis

All data were evaluated with a 2-tailed, unpaired Student t test or compared by 1-way ANOVA followed by the t-test and are expressed as mean±SD. A value of *P*≤0.05 was considered statistically significant.
