# Supplementary material for: Data on the involvement of Meox1 in balloon-injury-induced neointima formation of rats
Source: Data Brief. 2017 Nov 22;16:266–70. doi: 10.1016/j.dib.2017.11.061 (PMC5709307; doi:10.1016/j.dib.2017.11.061)
Supplement: Supplementary file 1 — Supplementary material [file mmc1.doc]

**Conflict of interest form**

The authors report no relationships that could be construed as a conflict of interest.
